# Towards the Development of Novel, Point-of-Care Assays for Monitoring Different Forms of Antioxidant Activity: The RoXsta^TM^ System

**DOI:** 10.3390/antiox13111379

**Published:** 2024-11-11

**Authors:** Robert J. Aitken, Alexandra Wilkins, Natasha Harrison, Kimia Kobarfard, Sarah Lambourne

**Affiliations:** 1 Centre for Reproductive Science, University of Newcastle, Callaghan, NSW 2308, Australia; alex.wilkins@newcastle.edu.au (A.W.); natasha.harrison@newcastle.edu.au (N.H.); kimia.kob@gmail.com (K.K.); sarah.lambourne@newcastle.edu.au (S.L.); 2 Hunter Medical Research Institute, New Lambton Heights, NSW 2305, Australia

**Keywords:** antioxidant measurement, ABTS, reactive oxygen species (ROS), spermatozoa, semen

## Abstract

(1) Background: This study set out to develop a series of simple, novel, rapid methods for assessing different forms of antioxidant activity. (2) Methods: An ABTS platform was used to engineer: (i) an electrochemical post-activation assay to assess free radical scavenging activity; (ii) an electrochemical pre-activation strategy to assesses the suppression of free radical formation; (iii) a horseradish peroxidase-mediated oxidation system to monitor hydrogen peroxide scavenging activity and (iv) a cumene peroxide-hematin system to determine the ability of samples to scavenge the mixture of organic peroxides and peroxyl and alkoxyl radicals generated in the presence of these reagents. Each assay was assessed against a panel of candidate antioxidant compounds to determine their relative activities and specificities. In addition, human semen samples were analyzed to determine how the results of these antioxidant assays correlated with semen quality. (3) Results: All 4 assays revealed dose-dependent antioxidant activity on the part of vitamin C, N-acetyl cysteine, hypotaurine, BSA, melatonin, glutathione, resveratrol and epigallocatechin gallate. The other compounds tested either completely lacked antioxidant activity or were only active in one of the assays. Using unfractionated human semen as an exemplar of biological fluids rich in antioxidants, the outputs from the individual assays were found to reflect different aspects of semen quality. When the data from all 4 assays were combined, accurate predictions were generated reflecting the importance of oxidative stress in defining semen quality as reflected by sperm count, seminal lipid aldehyde content, sperm DNA damage and free radical generation by the sperm mitochondria. (4) Conclusions: The methodologies described in this paper constitute the basis for rapid, point-of-care assessments of oxidative stress.

## 1. Introduction

Oxidative stress is known to play a key role in many biological and chemical processes, including food preservation, aging, hypertension, cancer, cardiomyopathy, cataract formation, diabetes, chronic lung disease, neurodegenerative disorders and a wide range of reproductive conditions including male and female infertility, endometriosis, PCOS, miscarriage and impaired offspring health [1,2]. One of the factors contributing to the significant amount of research in this area is the promise it holds for the therapeutic use of antioxidants to treat such conditions. The global antioxidants market is expected to grow from $4.13 billion in 2021 to $6.05 billion in 2028 at a compound annual growth rate of 5.61% over the period 2021–2028 [3]. Notwithstanding the potential in this area, the therapeutic value of antioxidant treatments is shrouded in uncertainty, for while tantalizing results have been obtained from time to time, the overall outcome of such therapeutic interventions has been generally disappointing [4,5,6]. Nowhere has this been truer than in the field of infertility.

Male infertility, in particular, has long been associated with the generation of oxidative stress [7]. Spermatozoa are vulnerable to such stress because they are richly endowed with substrates for free radical attacks, particularly polyunsaturated fatty acids and DNA [7,8]. In addition, the unique physical architecture of spermatozoa means that they are virtually devoid of internal cytoplasmic space in which to house antioxidant enzymes, such as glutathione peroxidase, superoxide dismutase and catalase, that protect most somatic cell types from oxidative attacks [8]. This lack of cytoplasm also means that the repair capacity of these cells is strictly limited, particularly with respect to the restoration of DNA damage and the maintenance of genetic integrity [9]. It also means that these cells are highly dependent on the antioxidant protection provided by the extracellular fluids in which these cells are bathed during their voyage through the male and female reproductive tracts. Given this vulnerability to oxidative stress, it may be surprising that spermatozoa are also professional generators of reactive oxygen species (ROS) that are used drive such critical physiological processes as capacitation and acrosomal exocytosis [10,11]. To fulfill this physiological need, spermatozoa are endowed with a variety of systems for generating ROS, including L-amino acid oxidases, NADPH oxidases and the mitochondrial electron transport chain, all of which are capable of generating excessive amounts of toxic oxygen metabolites under appropriate conditions [12,13,14]. In view of these considerations, it is not surprising that considerable effort has gone into conducting clinical trials to evaluate the impact of oral antioxidant therapy on sperm quality and fertility. These trials have generally produced mixed results, with a recent Cochrane analysis concluding that, overall, the current evidence is inconclusive and that “further large well-designed randomized placebo-controlled trials studying infertile men and reporting on pregnancy and live births are still required to clarify the exact role of antioxidants” [15].

The major problem with all antioxidant trials to date is that they have not selected patients on the basis of oxidative stress [16]. Infertility is a condition with multiple etiologies, not all of which involve some form of oxidant attack. If powerful antioxidant supplements are given to men who are not suffering from antioxidant depletion, there is a risk that a state of reductive stress may be created that may cause a further deterioration of semen quality via a variety of mechanisms including, ironically, the enhanced generation of ROS [17,18]. To avoid this problem, a simple, rapid, point-of-care assay for antioxidant availability is required that will provide an instantaneous appraisal of a patient’s redox status without the need to resort to complex, time-consuming laboratory methodologies.

The most commonly used method for assessing total antioxidant capacity (TAC) is a technique originally introduced by Rice-Evans’group [19] based on the oxidation of ABTS [2,2′-azino-bis (3-ethylbenzothiazoline-6-sulfonic acid)]. In this assay, ABTS is oxidized, using either a combination of metmyoglobin and hydrogen peroxide or a chemical oxidant such as potassium persulphate, to generate the ABTS cation radical (ABTS•^+^), which is blue-green in color. Subsequent addition of an antioxidant leads to discoloration of the ABTS•^+^, largely via an electron transfer mechanism, the degree of discoloration reflecting the level of antioxidant activity.

In this article, we describe several adaptations of the ABTS assay to provide rapid assessments of antioxidant activity and compare their responsiveness to a broad range of candidate antioxidants. We also explore the usefulness of these assays in a diagnostic context by examining their relationships with human semen quality.

## 2. Materials and Methods

### 2.1. Clinical Materials

The major clinical material used in the development of these antioxidant assays was human semen. Ethical approval from the University of Newcastle human research ethics committee and State Government was secured for the use of human semen samples in this study (H-2013-0319 and 200621). The samples used in this analysis were collected from 27 unselected, normozoospermic males of unknown fertility status, aged 18–76 years, who were members of the University of Newcastle semen donor panel. The fundamental attributes of semen quality (mean ± standard error) for this donor cohort on the day of analysis were semen volume (3.2 ± 0.3 mL), normal morphology (6.6 ± 0.6%), motility (43.2 ± 3.8%), sperm concentration (91.0 ± 14.4 × 10^6^/mL) and vitality (79.3 ± 2.1%). Donor samples were provided to the laboratory following 48 h of sexual abstinence. After allowing at least 30 min for liquefaction to occur, the spermatozoa were separated by 3 cycles of centrifugation and resuspension at 500× *g* for 5 min using HEPES-buffered Biggers-Whitten-Whittingham medium (BWW) [20] supplemented with 1 mg/mL polyvinyl alcohol. The spermatozoa were finally resuspended in BWW at a concentration of 10 × 10^6^/mL.

### 2.2. Chemical Materials

All chemicals were purchased from Sigma-Aldrich (St Louis, MO, USA). For the free radical scavenging assay, the antioxidants assessed in this study were dissolved in 0.85% saline (vitamin C, glutathione, N-acetyl cysteine, hypotaurine, carnitine, taurine, spermine, myoinositol and epigallocatechin gallate). Additional compounds were dissolved in dimethyl sulfoxide (DMSO), including melatonin, lipoic acid, coenzyme Q10 and resveratrol, or ethanol in the case of Trolox. In general, the stock solutions were made up at a concentration of 5 mM, although 70 mM stocks were used for log dose–response studies. In the pre-activation version of the ABTS [2,2′-azino-bis (3-ethylbenzothiazoline-6-sulfonic acid)] assay, where the focus was on the inhibition of free radical creation, DMSO could not be used, because this compound was extremely active in suppressing ABTS•^+^ formation. As alternatives, some compounds were dissolved in ethanol (melatonin, lipoic acid, resveratrol and Trolox) while co-enzyme Q10 was dissolved in N, N-dimethylformamide (DMF). For the hydrogen peroxide and lipid peroxide scavenging assays, the compounds were solubilized as described for the free radical scavenging assay.

### 2.3. Assessment of Sperm Quality and Function

#### 2.3.1. Sperm Counts and Sperm Motility

Sperm counts in semen were determined using a Nucleocounter (ChemoMetec, Allerod, Denmark), while the quality of sperm movement was assessed with a CASA system (Hamilton Thorne, IVOS II, Beverly, MA, USA) using the following settings: minimum total count 200, kinematics: progressive STR (%) 80, progressive VAP (µm/s) 25, static VAP (µm/s) 0, static VSL (µm/s) 0.

#### 2.3.2. Sperm DNA Fragmentation

DNA Integrity was studied using the Halo assay on spermatozoa that had been resuspended in BWW and snap frozen in liquid nitrogen. This laboratory assay involved setting the spermatozoa in 0.65% agarose on slides which were then immersed in 0.08 M HCl for 7 min, followed by 100 mM DTT in Tris buffer 1 (4.84 g of Tris, 10 mL of 10% SDS, 10 mL of 0.5 M EDTA made up to 100 mL with MilliQ; pH 7.5) for 10 min. The slides were then immersed in Tris buffer 2 (4.84 g Tris, 11.69 g NaCl, 10 mL 10% SDS made up to 100 mL with MilliQ; pH 7.5) for 5 min, followed by immersion in Tris-Boric Acid-EDTA Buffer (TBE: 5.4 g Tris, 2.75 g boric acid, 2 mL of 0.5 M EDTA made up to 100 mL with MilliQ; pH 7.5) for 2 min. The slides were subsequently passed through increasing strengths of ethanol (70%, 90% and 100% ethanol), allowing 2 min for each step. They were then air dried and stained with 4′,6-diamidine-2′-phenylindole dihydrochloride (DAPI, Sigma-Aldrich, St Louis, MO, USA) solution for 10 min (1 µL of DAPI per 2 mL of phosphate-buffered saline [PBS: pH 7.4, 137 mM NaCl, 2.7 mM KCl, 8 mM Na2HPO4, and 2 mM KH2PO4], generating a final DAPI concentration of 1.8 µg /mL). The slides were finally rinsed with PBS, and 30 µL of Mowiol (Sigma-Aldrich, St Louis, MO, USA) were added and coverslips applied. For scoring purposes, the cells were classified into five categories: large halo, medium halo, small halo, no halo or degraded sperm. The percentage of DNA-damaged spermatozoa was given by the percentage of cells falling into the small halo, no halo and degraded categories, as recommended [21].

#### 2.3.3. Lipid Aldehyde Formation in Semen

The measurement of malondialdehyde levels in unfractionated human semen samples was undertaken using 1-methyl-2-phenylindone [22]. For this assay, 15.4 mM 1-methyl-2-phenylindone was made up in acetonitrile: methanol (3:1). Iron (64 mM ferrous sulfate) and ascorbate (1M) were combined in a 1:1 ratio and 2 µL of this promoter was added to 98 µL of unfractionated semen before the mixture was diluted with 100 µL acetonitrile: methanol (3:1). Either 650 µL 1-methyl-2-phenylindone or the same volume of acetonitrile: methanol (as a blank) was then added to this mixture. The solution was subsequently acidified with the addition of 150 µL 32% HCl and incubated for 1 h at 45 °C. Following incubation, the samples were clarified by centrifugation at 1500× *g* for 5 min and finally read at 586 nm in a plate reader (SPECTROstar Nano, BMG Labtech, Ortenberg, Germany). The standard used for this assay was 1,1,3,3-tetramethoxypropane (Sigma-Aldrich, St Louis, MO, USA).

#### 2.3.4. Mitochondrial Reactive Oxygen Species

ROS generation by the mitochondria of isolated spermatozoa was detected by flow cytometry using MitoSOX™ Red dye (MSR, Molecular Probes). This reagent was prepared at a dilution of 1 µL of a 5 mM MSR stock solution in DMSO in 249 µL BWW. Sytox^®^ Green dye (SyG, Molecular Probes) was purchased as a 5 mM stock solution in DMSO and added at a dilution of 1 µL 12.5 mM SyG in 249 µL BWW in order to differentiate live and dead cells (required because trace amounts of ethidium in the MSR probe can bind directly to the nuclei of dead cells with compromised plasma membrane integrity). As a positive control, arachidonic acid (AA; Sigma Aldrich, St Louis, MO, USA) was added at a concentration of 50 µM. Once stained with MSR and SyG solutions, the spermatozoa were incubated at 37 °C for 15 min before centrifugation and resuspension in BWW. The results were ultimately expressed as the percentage of live MSR positive cells/all live cells [12]. Flow cytometry analyses were conducted on a FACSCalibur flow cytometer (Becton Dickinson, Franklin Lakes, NJ, USA) with a 488 nm argon ion laser. Forward scatter and side scatter measurements were taken to generate a scatter plot, which was used to gate for sperm cells only, excluding any larger contaminating cells. All data were analyzed using BD FACSDiva V8.01 software (Becton Dickinson, Franklin Lakes, NJ, USA).

### 2.4. Measurement of Antioxidant Activity

In this study, we developed assays to assess 4 different aspects of antioxidant activity: (1) an electrochemical post-activation assay designed to assess free radical scavenging activity, (2) an electrochemical pre-activation assay designed to assess the ability of a sample to interfere with free radical formation, (3) a horseradish peroxidase (HRP)-based assay to assess hydrogen peroxide scavenging activity and (4) a hematin and cumene hydroperoxide-based assay to determine the ability of a given sample to scavenge organic peroxides, as well as the peroxyl and alkoxyl radicals generated by this combination of reagents. All these assays were developed from the same fundamental platform in which the colorless phenothiazine compound, ABTS, is oxidized to produce the radical cation (ABTS•^+^), which is blue-green and can be monitored spectrophotometrically at 734 nm [19]. Typically, this assay is calibrated with a water-soluble vitamin E analog (Trolox) and the results expressed in Trolox equivalents; in this form, the assay is widely known as the TEAC (Trolox Equivalent Antioxidant Capacity) assay [23,24,25].

#### 2.4.1. Post-Activation Free Radical Scavenging Activity

**Figure 1:**
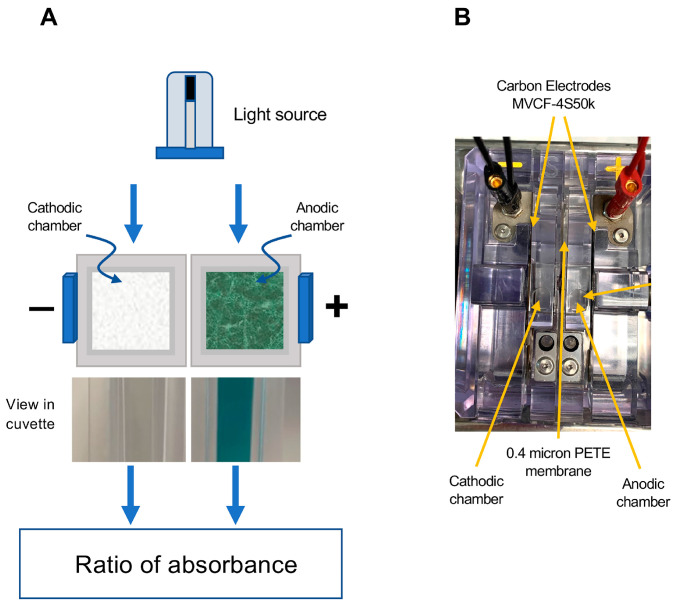
Diagrammatic depiction of the electrochemical cell used to activate ABTS for the free radical scavenging/formation assay. (**A**) Following the passage of current, ABTS is oxidized to the blue-green ABTS•^+^ cation radical in the anodic chamber, which can be read at 734 nm. The reducing environment of the cathodic chamber does not allow this chemistry to occur, so it serves as a control channel, permitting the assessment of interference due to turbidity. The chambers are separated by a semipermeable 0.4 µm PETE (Polyester Track Etched) membrane to allow the passage of current. (**B**) Aerial perspective of the actual electrochemical device showing the position of the anodic and cathodic chambers, the MVCF-4S50k electrodes and the chamber separation membrane.

The fundamental concept that we have employed in developing this assay is to achieve ABTS activation using an electrochemical cell with anodic and cathodic chambers separated by a semipermeable membrane. The fundamental design of this electrochemical cell is depicted in Figure 1. It comprised 2 cells separated by a 0.4 µm PETE (Polyester Track Etch) membrane (Sterlitech, Auburn, WA, USA) and connected to MVCF-4S50K carbon electrodes (CAPLINQ, Livonia, MI, USA) to permit the passage of a current. In such a device, the ABTS becomes oxidized in the anodic chamber, generating a deep blue-green color while the reducing environment provided by the cathodic chamber serves as a negative control that can be used to correct subsequent spectrophotometric readings for the turbidity often encountered in biological samples such as human semen (Figure 1).

For this assay, the parent ABTS solution was made up in 50 mM phosphate buffer at pH 4.8 (20.1 mg sodium phosphate dibasic heptahydrate, 1.71 g sodium dihydrogen phosphate monohydrate and 4.5 g sodium chloride in 500 mL MilliQ water). An acid pH was chosen for this assay because it helped stabilize the ABTS•^+^ radical and generated maximal levels of absorbance [26]. Where indicated, the pH of the phosphate buffer was raised to 7.5 to see whether an elevated pH might enhance the antioxidant activity of some compounds by promoting deprotonation of hydroxyl groups and facilitating electron transfer reactions. For the routine assay, 4 mL of buffer containing ABTS (100 µM) was pipetted into the chambers of the electrochemical cell and activated by passing a current (45 V and 10 mA) for 20 s; at the end of this time, the fluid in the anodic chamber had turned blue-green, while the cathodic chamber remained colorless. After 3 min, one mL aliquots of the cathodic and anodic ABTS solutions were then pipetted into cuvettes for spectrophotometry (SPECTROstar Nano, BMG Labtech, Ortenberg, Germany). After an initial reading at 734 nm, 15 µL aliquots of semen or antioxidant were added to each cuvette, mixed and allowed to stand for 5 min at room temperature. At the end of this period, the absorbance was measured again. The change in absorbance, corrected for the turbidity reading recorded in the cathodic chamber, was then calculated. Standards were created from a 5 mM Trolox stock solution (6.26 mg in 5 mL of 50% ethanol) so that the final concentrations following the addition of 15 µL to 1 mL of activated ABTS•^+^ ranged from 0 to 30 µM.

#### 2.4.2. Pre-Activation Assessment of Ability to Inhibit Free Radical Formation

This assay was essentially the same as the free radical scavenging assay, except that the order in which the reagents were added to the electrochemical cell was reversed. In this case, a solution was prepared by mixing 5.4 mL of 100 µM ABTS in phosphate buffer with 100 µL of sample and pipetted into the anodic and cathodic chambers of the electrochemical cell. When the assay was used for assessing human semen samples, 16.5 µL of the latter were mixed with 183.5 µL of 50 mM phosphate buffer (pH 4.8) to create an 8.25% dilution and 100 µL of this mixture was added to 5.4 mL of 100 µM ABTS as indicated above (667-fold final dilution). When assessing constituents of the antioxidant panel, the dilution factor was 100 µL into 5.4 mL ABTS (55-fold dilution). After exactly 1.5 min, electric current was run through the cell (20 s at 45 V and 10 mA) to achieve activation of the ABTS in the anodic chamber, with the cathodic chamber serving as a passive control for sample turbidity. Immediately after the passage of current, the contents of the anodic chamber were agitated by pipetting up and down 10× and the cell was left for 5 min to allow the activation process to be completed. The contents of the anodic and cathodic chambers were then transferred to a spectrophotometer and the absorbance measured at 734 nm (SPECTROstar Nano, BMG Labtech, Ortenberg, Germany). The corrected absorbance was then calculated. In running this assay, it was found that the ability of Trolox to inhibit ABTS•^+^ radical formation was less effective and less consistent than vitamin C, so the latter was used to create the corresponding standard curves.

#### 2.4.3. Assessment of Ability to Scavenge Hydrogen Peroxide

This assay was based on the ability of test samples to inhibit the oxidation of ABTS to the ABTS•^+^ colored cation radical, via by the action of hydrogen peroxide in the presence of HRP. For this reaction, the buffer employed was a 50 mM phosphate buffer at pH 6.5 (1.197 g sodium phosphate dibasic heptahydrate and 1.109 g sodium phosphate monobasic monohydrate made up in 250 mL Milli Q water). Using this buffer, the following reagents were prepared: (i) 1 mg/mL HRP, (ii) 10 mM ABTS, (iii) 300 µM hydrogen peroxide and (iv) diluted test sample comprising 7.5 µL of semen in 500 µL phosphate buffer. The final reaction mixture contained 735 µL buffer, 15 µL ABTS (150 µM), 100 µL antioxidant/semen sample (0.15% semen) and 50 µL HRP (0.05 mg/mL). The reaction was activated by the addition of 100 µL hydrogen peroxide (30 µM) and incubated at room temperature for 10 min to allow the formation of the colored radical cation. The absorbance was finally read at 734 nm in a plate reader (SPECTROstar Nano, BMG Labtech, Ortenberg, Germany). Controls for sample turbidity were created by omitting ABTS, while a no-sample control was created by replacing the addition of diluted semen with distilled water.

Trolox was used to calibrate this assay, and the results are expressed in Trolox equivalents. Standards were created from a 5 mM Trolox stock solution so that the final concentrations ranged from 0 to 20 µM. When assessing the constituents of the antioxidant panel, the dilution was 100 µL into 900 µL (10-fold dilution); however, when assessing semen samples, an additional dilution (667-fold) was required, given the extremely high level of antioxidant activity expressed by this fluid.

#### 2.4.4. Assessment of Ability to Scavenge Organic Peroxide

This assay was similar to the hydrogen peroxide scavenging assay, except that horseradish peroxidase was replaced by hematin [27]. The latter was prepared at 0.5 mg/mL in DMSO. The buffer for this assay was the same 50 mM phosphate buffer (pH 6.5) as employed for the hydrogen peroxide scavenging assay. The constituents of the reaction mixture (final concentrations in brackets) were 675 µL buffer, 25 µL ABTS (250 µM), 100 µL antioxidant or diluted semen sample (0.15% semen) and 100 µL hematin (0.05 mg/mL). The reaction was activated by the addition of 100 µL, 1 mM cumene hydroperoxide (100 µM) and incubated at room temperature for 20 min before the absorbance was determined at 734 nm using a plate reader as described above. ABTS was replaced with phosphate buffer in order to create a turbidity control, while a no-sample control was created by replacing the test material with distilled water. The results were expressed in Trolox equivalents.

### 2.5. Statistical Analysis

All data were analyzed using JMP Pro software (Version 17, SAS Institute, Cary, NC, USA) employing simple or multiple linear regression analysis and ANOVA, while comparisons between group means were conducted using the Tukey–Kramer HSD post hoc test. The normality of the data distribution was assessed using the Shapiro–Wilk procedure and, where necessary, data were normalized by square root transformation as indicated.

## 3. Results

### 3.1. Post-Activation Free Radical Scavenging Assay

**Figure 2:**
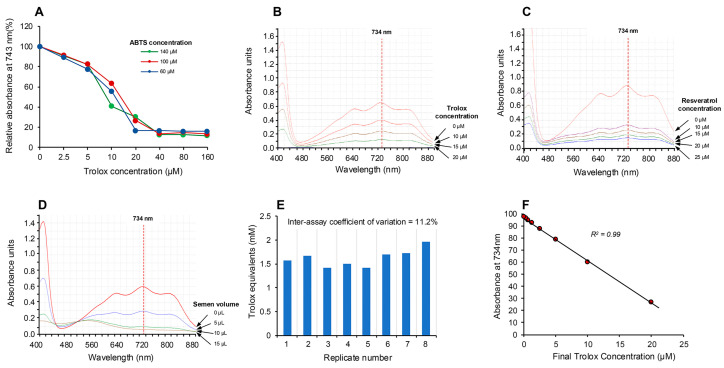
Electrochemical activation of ABTS and its application in the assessment of free radical scavenging activity. (**A**) Electrochemical activation of ABTS for 20 s generated the colored ABTS•^+^ radical cation, which absorbed intensely at 734 nm but could be suppressed by post-activation addition of the antioxidant Trolox, in a manner that was not significantly changed by ABTS concentrations ranging from 60 to 140 µM. (**B**) The absorption spectrum generated by ABTS•^+^ showing the peak at 734 nm and the dose-dependent suppression achieved by Trolox. (**C**) A similar dose-dependent suppression of ABTS•^+^ absorption at 734 nm achieved with resveratrol. (**D**) Using unfractionated human semen as a rich biological source of antioxidant activity, a dose-dependent decrease in absorption at 734 nm was achieved. (**E**) Repeated analysis of the same human semen sample generated an inter-assay coefficient of variation of 11.2% with this post-activation, electrochemical ABTS assay (*n* = 8). (**F**) A detailed dose–response analysis with Trolox demonstrated that the suppression of ABTS•^+^ absorption was linear over the final concentration range, 0.31–20 µM.

Using the electrochemical cell depicted in Figure 1 and Trolox as a model antioxidant, a brief 20 s application of current was found to generate the colored ABTS•^+^ cation radical within the ensuing 5 min. This radical could be returned to its non-colored parent state by Trolox in a dose-dependent manner over the 5–25 µM dose range (final concentration) and was not significantly impacted by ABTS concentrations extending from 60 to 140 µM (Figure 2A).

The absorption spectrum (Figure 2B) exhibited peaks at 415, 645, 734 and 815 nm [28], and, in keeping with previous studies, we selected 734 nm as the optimum wavelength to monitor the assay, since it minimizes interference due to other absorbing materials and turbidity [29,30]. The ability of this system to rapidly assess antioxidant activity was subsequently confirmed using the classical antioxidants, Trolox and resveratrol [19,24], which induced the anticipated dose-dependent suppression of absorption at 734 nm as depicted in Figure 2B,C. Using this approach, we then assessed the ability of human semen, a known source of potent antioxidant activity [31], to suppress the electrochemically generated ABTS•^+^ radical signal and observed a clear dose-dependent suppression (Figure 2D). The robustness of the assay was indicated by multiple (10×) measurements of a single standard containing 10 µM Trolox, which generated an intra-assay coefficient of variation of 4.36%. The inter-assay coefficient of variation was determined by analyzing aliquots of a single human semen sample on 8 independent occasions; this analysis generated an inter-assay coefficient of variation of 11.2% (Figure 2E). Using this electrochemical system, a Trolox standard curve was created which indicated that the response was linear over the range 0.31–20 µM and that the system could readily detect 5 µM Trolox in the final reaction mixture (Figure 2F).

**Figure 3:**
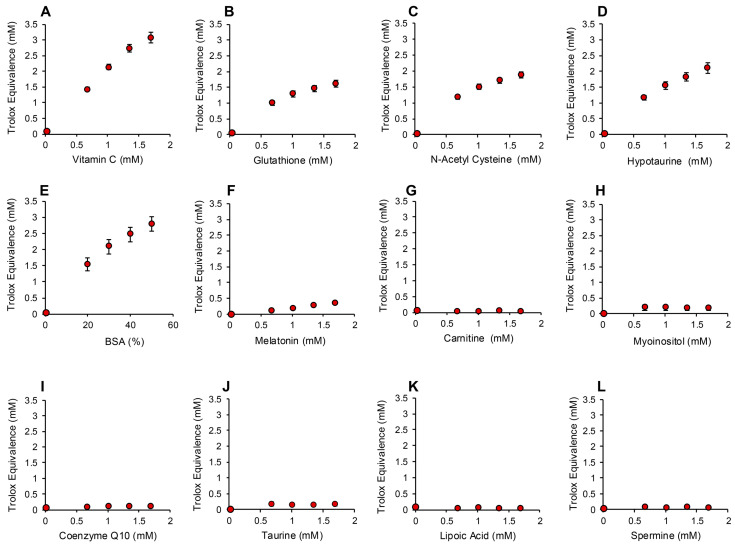
Use of the post-activation electrochemical ABTS assay for free radical scavenging activity to determine the ability of candidate antioxidants to suppress the ABTS•^+^ signal. Dose-dependent analyses were conducted for (**A**) vitamin C, (**B**) glutathione, (**C**) N-acetyl cysteine, (**D**) hypotaurine, (**E)** BSA, (**F**) melatonin, (**G**) carnitine, (**H**) myoinositol, (**I**) coenzyme Q10, (**J**) taurine, (**K**) lipoic acid and (**L**) spermine. All results are presented as the concentration of reagent in Trolox equivalents in the original 15 µL inoculant into the medium. All data points represent the mean ± SE of 3 independent replicates.

**Figure 4:**
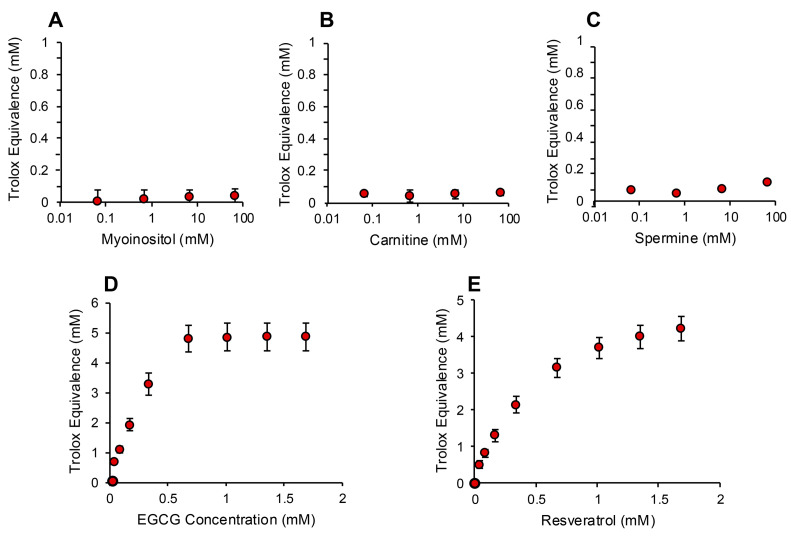
Assessment of antioxidant activity using the post-activation ABTS assay. Log dose response analyses for (**A**) myoinositol, (**B**) carnitine and (**C**) spermine failed to reveal any free radical scavenging activity. In contrast, both polyphenols, (**D**) epigallocatechin gallate (EGCG) and (**E**) resveratrol, exhibited such powerful antioxidant activity that they reached a plateau at concentrations above 0.676 mM (10 µM final concentration). All data points represent the mean ± SE of 3 independent replicates.

In order to determine whether the electrochemical activation of ABTS generated results that are representative of antioxidant activity, we examined the performance of this post-activation assay in the presence of a number of compounds that are reputed to possess such properties. The results of this analysis are presented in Figure 3 in terms of the actual concentration of reagent in the 15 µL inoculated into the assay; the final concentration following dilution with the assay reagents requires division by 67.6 (equivalent to 10–25 µM). In addition to Trolox, compounds known to possess free radical scavenging activity, such as vitamin C, glutathione, N-acetyl cysteine, bovine serum albumin (BSA) and hypotaurine, all exhibited clear dose-dependent activity in this assay (Figure 3A–E). Melatonin was also active as an antioxidant, but to a much lesser degree (Figure 3F). However, no dose-dependent activity could be detected for carnitine, myoinositol, coenzyme Q10, taurine, lipoic acid or spermine (Figure 3G–L). To ensure that this was a qualitative rather than a quantitative difference, log dose responses were conducted for myoinositol, carnitine and spermine (Figure 4A–C), and still no free radical-scavenging activity was detected at doses of up to 67.6 mM (equivalent to a final concentration of 1 mM once the stock had been diluted into the reaction buffer). In addition, this experiment was repeated at a pH of 7.4 to determine whether enhanced deprotonation of the hydroxyl groups in the case of carnitine and myoinositol would facilitate electron transfer. However, even at this pH, none of these putative antioxidants exhibited significant free radical scavenging activity (Figure S1, Supplementary Materials). In complete contrast, the polyphenols epigallocatechin gallate (EGCG) and resveratrol were such efficient free radical scavengers at pH 4.8 that they saturated the assay at low concentrations (final concentrations of 0.01 mM and 0.025 mM for EGCG and resveratrol, respectively) (Figure 4D,E).

**Figure 5:**
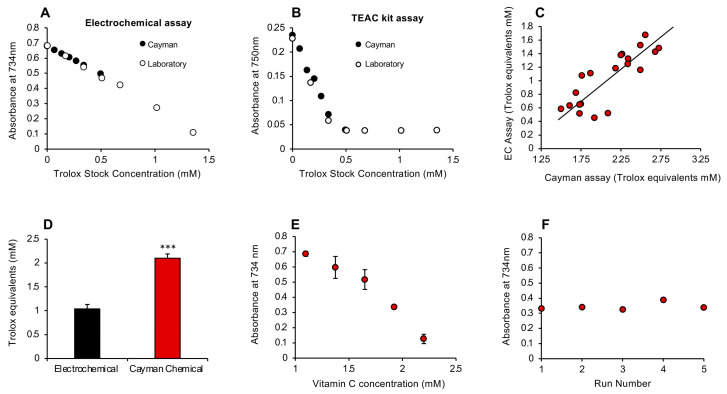
Comparison of the post-activation electrochemical free scavenging assay with a commercial kit manufactured by Cayman Chemicals and subsequent development of a pre-activation assay. (**A**) Using the electrochemical assay, the standards provided by Cayman (closed circles) or constructed in the laboratory (open circles) were superimposable and linear. (**B**) When the commercial TEAC (Trolox Equivalent Antioxidant Capacity) kit was used for these assessments, the standard curves were again linear and superimposable at the lower doses, but the Cayman assay lost sensitivity above 0.5 mM Trolox. (**C**) A comparison of the ability of the post-activation electrochemical (EC) and Cayman assays to measure the antioxidant content of human semen revealed a highly significant correlation (r^2^ = 0.68; *p* < 0.001) between these methods of assessment. (**D**) However, the Cayman assay reported almost twice as much antioxidant activity in semen as the electrochemical equivalent (*** *p* < 0.001; *n* = 20), possibly because the former is a pre-activation assay whereby test sample is added prior to ABTS oxidation, thereby enabling the assay to reflect the inhibition of free radical formation as well as free radical scavenging. (**E**) A pre-activation version of the electrochemical assay was therefore developed and shown to generate a linear standard curve with vitamin C. X-axis presents the concentration of vitamin C added to the reaction mixture; final concentration was 20–40 µM. All data points represent the mean ± SE of 3 independent replicates. (**F**) This pre-activation electrochemical assay was extremely stable, generating an inter-assay coefficient of replication of 0.001% on 5 independent replicate analyses of a solution containing 35 µM vitamin C.

To determine how this rapid antioxidant assay compared with the conventional ABTS assay marketed by the Cayman Chemical Company (Ann Arbor, MI, USA), we took the Trolox standards from each assay and analyzed them using either the electrochemical activation protocols described in this study or the Cayman kit. This analysis indicated that, while the results for Trolox standards were superimposable regardless of which assay was used, the electrochemical activation assay was clearly linear over a much greater range of concentrations (Figure 5A,B).

Despite their apparent equivalence, an interesting difference between the Cayman procedure and the post-addition electrochemical system is the order in which reagents are added. With the latter, the passage of an electric current is used to oxidize ABTS to the ABTS•^+^ radical in the anodic chamber, and then the assay looks at the ability of a given test sample to scavenge the radical species so generated. With the Cayman assay, the test sample is added first, and then the ABTS is oxidized using hydrogen peroxide in concert with metmyoglobin. It therefore measures the ability of a test substance to interfere with free radical formation as well as scavenge any radicals subsequently formed. This procedural difference may account for the fact that, when the electrochemical assay and Cayman kit were compared with a group of 20 semen samples, the levels of antioxidant activity recorded were highly correlated with each other (r^2^ = 0.68; *p* < 0.001; Figure 5C); however, the values generated by the Cayman assay were significantly higher (*p* < 0.001) than those secured with the post-activation electrochemical assay (Figure 5D). To determine whether this difference reflected the ability of the Cayman assay to capture the suppression of radical formation as well as free radical scavenging, we revised the electrochemical assay to incorporate a pre-activation strategy, whereby the test sample would be added prior to the application of electric current.

### 3.2. Pre-Activation Assay for the Inhibition of Free Radical Formation

A pre-activation strategy was therefore developed and shown to respond to antioxidant addition. Both vitamin C and Trolox were tested as standards for this assay, and the former was found to be both more sensitive and more reliable than Trolox, which was poorly active in this system (Figure S2, Supplementary Materials). Vitamin C, however, generated a clear dose-dependent suppression of ABTS•^+^ radical formation and an extremely low inter-assay coefficient of variation (0.001%) on 5 independent assays conducted over a period of 3 days (Figure 5E,F).

**Figure 6:**
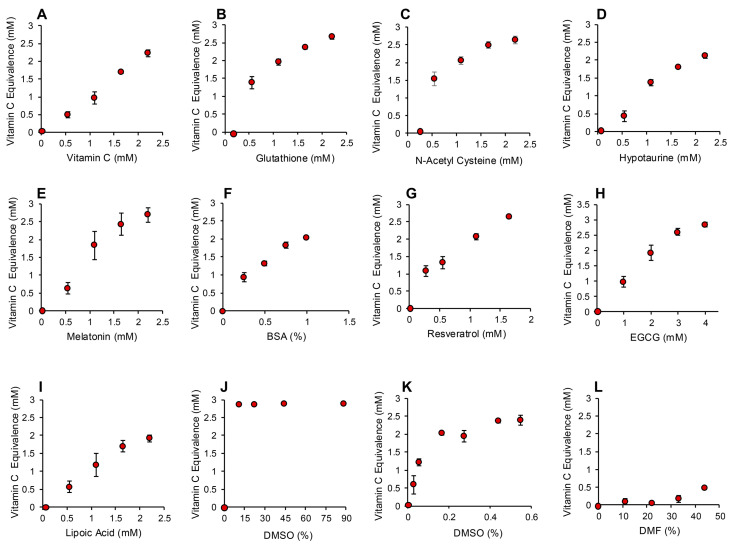
Pre-activation electrochemical ABTS assay to determine the ability of antioxidants to suppress formation of the ABTS•^+^ signal. Dose dependent analyses were conducted for (**A**) vitamin C, (**B**) glutathione, (**C**) N-acetyl cysteine, (**D**) hypotaurine, (**E**) melatonin, (**F**) BSA, (**G**) resveratrol (**H**) EGCG, (**I**) lipoic acid, (**J**) DMSO, high dose, (**K**) DMSO low dose and (**L**) DMF. All results are presented as the concentration of reagent in the original solution. All data points represent the mean ± SE of 3 independent replicates.

The application of this assay to the same range of antioxidants that had been tested in the post-activation assay described above generated very similar results. Again, compounds such as carnitine, myoinositol, co-enzyme Q10, taurine and spermine exhibited no detectable antioxidant activity under the conditions of this assay (Figure S2, Supplementary Materials). Trolox was also inactive at the doses used for screening the antioxidant panel (10–40 µM final concentration), although higher concentrations were effective (Figure S2, Supplementary Materials). Antioxidants such as vitamin C, glutathione, N-acetyl cysteine, hypotaurine, melatonin, BSA, resveratrol and EGCG were highly active in this version of the assay (Figure 6A–H and Figure S2, Supplementary Materials). Interestingly, lipoic acid, which had been inactive in the post-activation electrochemical assay, was intensely active in this pre-activation system, suggesting an important role for this compound in the suppression of free radical formation (Figure 6I). Another major surprise with the pre-activation system was the activity displayed by DMSO, which had been inactive in the post-activation model. Concentrations of DMSO in excess of 10% completely saturated this version of the assay, preventing all formation of the ABTS•^+^ radical. A more restricted standard curve revealed that as little as 0.0275% DMSO (0.0005% final concentration) could suppress ABTS•^+^ radical formation in this assay, suggesting that this compound is an extremely effective inhibitor of free radical formation. For this reason, we had to use DMF or ethanol as solvents in this assay because they possessed much more limited antioxidant activity (Figure S2, Supplementary Materials and Figure 6L).

### 3.3. Assay for Hydrogen Peroxide Scavenging

Another version of the pre-activation antioxidant assay was then developed that focused on the ability of hydrogen peroxide to induce ABTS oxidation when added to a mixture containing diluted test sample, ABTS and HRP. With this assay, any factors in the test sample that scavenged hydrogen peroxide would have a proportional impact on ABTS oxidation to the radical cation.

**Figure 7:**
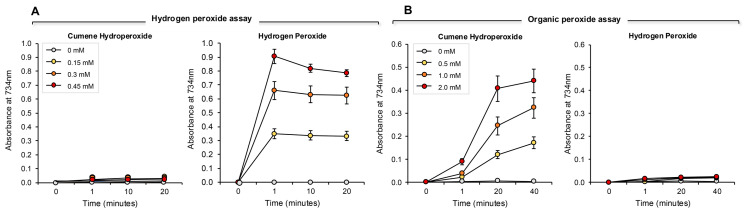
Development of ABTS assays for hydrogen- and organic- peroxides. (**A**) The hydrogen peroxide assay was completely unresponsive to 0.15–0.45 mM cumene hydroperoxide over a time scale of 20 min. However, the assay responded extremely rapidly to the presence of the same doses of hydrogen peroxide, such that a clear dose–response was evident at the earliest time point assessed (~1 min). Once generated, the ABTS•^+^ radical was relatively stable, although some deterioration on the signal was evident over 20 min with the highest standard assessed. (**B**) Conversely, the cumene hydroperoxide assay was extremely sensitive to 0.5–2.0 mM organic peroxide, but did not respond at all to hydrogen peroxide over the 40 min incubation period. Unlike the hydrogen peroxide assay, the generation of an ABTS•^+^ signal following exposure to cumene hydroperoxide and hematin was not instantaneous, but evolved gradually. The results represent the mean ± SE of 3 independent experiments.

**Figure 8:**
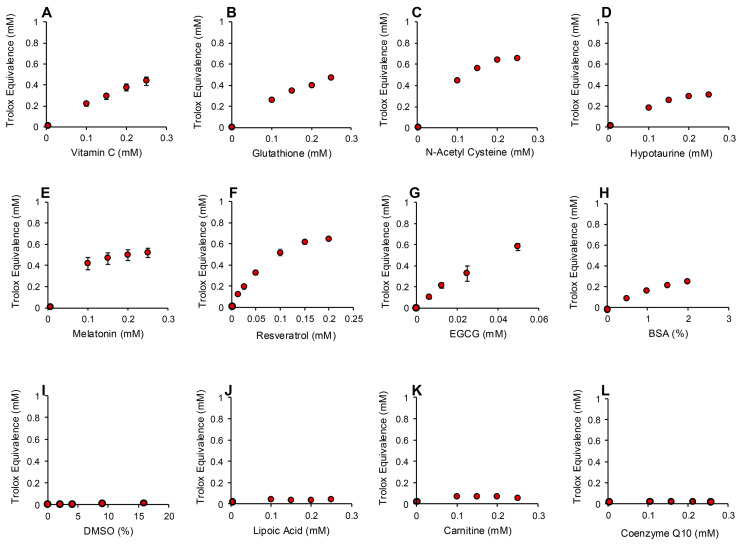
Use of ABTS and HRP to monitor hydrogen peroxide scavenging activity of putative antioxidants. Dose-dependent analyses were conducted for (**A**) vitamin C, (**B**) glutathione, (**C**) N-acetyl cysteine, (**D**) hypotaurine, (**E**) melatonin, (**F**) resveratrol (**G**) EGCG, (**H**) BSA, (**I**) DMSO, (**J**) lipoic acid, (**K**) carnitine and (**L**) co-enzyme Q10. All results are presented as the concentration of reagent in the original solution. All data points represent the mean ± SE of 3 independent replicates.

The addition of increasing amounts of hydrogen peroxide to this system generated a proportional, dose-dependent increase in ABTS•^+^ radical formation that could be assessed by measuring absorption at 734 nm. The use of HRP made this assay both rapid (maximal activity within 1 min) and specific for hydrogen peroxide; organic peroxides such as cumene hydroperoxide (a lipid peroxide mimetic) were minimally active in this system (Figure 7A). Using this assay, we then determined the hydrogen peroxide scavenging activity of the antioxidants previously analyzed with the pre- and post- activation electrochemical assay. The results of this analysis are presented in Figure 8 and Figure S3 (Supplementary Materials). Dose-dependent antioxidant activity was again observed with vitamin C, glutathione, N-acetyl cysteine, hypotaurine, melatonin, resveratrol, EGCG and BSA (Figure 8A–H). In contrast, DMSO, lipoic acid, carnitine, coenzyme Q10, taurine, spermine, myoinositol and ethanol were all devoid of significant hydrogen peroxide scavenging activity in this assay (Figure 8 and Figure S3, Supplementary Materials).

### 3.4. Assay for Organic Peroxide, Peroxyl and Alkoxyl Radical Scavenging

**Figure 9:**
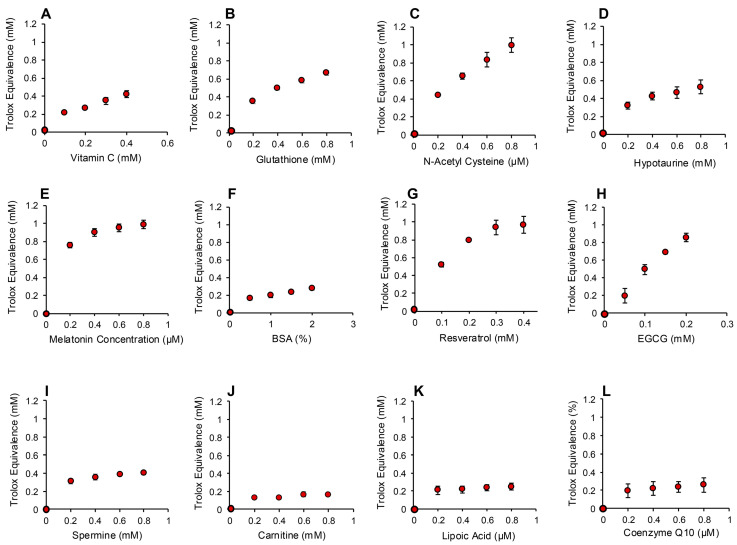
Use of the ABTS and hematin to monitor the scavenging activity of putative antioxidants towards organic peroxide, peroxyl and alkoxyl radicals. Dose-dependent analyses were conducted for (**A**) vitamin C, (**B**) glutathione, (**C**) N-acetyl cysteine, (**D**) hypotaurine, (**E**) melatonin, (**F**) BSA (**G**) resveratrol (**H**) EGCG, (**I**) spermine, (**J**) carnitine, (**K**) lipoic acid and (**L**) co-enzyme Q10. All results are presented as the concentration of reagent in the original solution. All data points represent the mean ± SE of 3 independent replicates.

The ABTS platform was also used to gauge the ability of test compounds and complex biological fluids to scavenge the mixture of hydroperoxides (ROOH), peroxyl (ROO) and alkoxyl (RO) radicals created via the interaction between cumene hydroperoxide and hematin [32] (Figure 7B). The response of ABTS to hematin/cumene peroxide-induced oxidation was slower and less sensitive than the HRP/hydrogen peroxide reaction; however, it was relatively specific for the organic peroxide, generating little response with hydrogen peroxide (Figure 7B). Using this procedure, the same group of antioxidants were scanned for their ability to scavenge the mixture of organic peroxide and free radical species responsible for ABTS oxidation in the presence of hematin and organic peroxide. In this case, we again saw significant dose-dependent antioxidant activity on the part of vitamin C, glutathione, N-acetyl cysteine, hypotaurine, melatonin, BSA, resveratrol and EGCG (Figure 9A–H). Spermine also exhibited some scavenging activity that plateaued at 0.3 mM Trolox equivalents (Figure 9I). The other compounds tested also exhibited a very low level of scavenging activity that was not dose-dependent and plateaued at 0.2 mM Trolox equivalents including carnitine, lipoic acid, coenzyme Q10 (Figure 9J–L), taurine, myoinositol, DMSO and ethanol (Figure S4, Supplementary Materials).

### 3.5. Analysis of Human Semen

**Figure 10:**
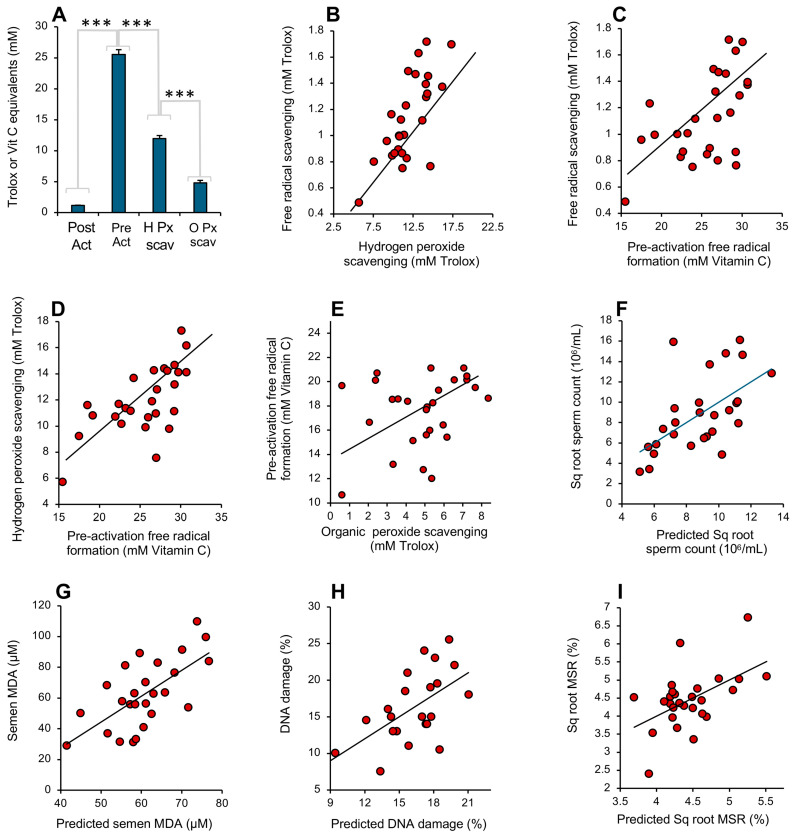
Performance of the antioxidant assays in the assessment of human semen. (**A**) Analysis of the antioxidant levels recorded in semen samples donated by 27 randomly selected males. All groups are significantly different from each other by ANOVA (*** *p* < 0.001). Note the extremely high level of activity recorded by the pre-activation free radical formation assay, equivalent to a mean of 25 mM vitamin C equivalents. (**B**) Correlation between the hydrogen peroxide scavenging assay and post-activation free radical scavenging assay (*r*^2^ = 0.52; *p* < 0.001). (**C**) Correlation between post-activation, free radical scavenging assay and the pre-activation assessment of free radical formation (*r*^2^ = 0.30; *p* < 0.01). (**D**) Correlation between the hydrogen peroxide scavenging assay and the pre-activation assessment of free radical formation (*r*^2^ = 0.43; *p* < 0.001). (**E**) Relationship between the assessment of organic peroxide scavenging activity and the pre-activation assessment of free radical formation (*r*^2^ = 0.08; NS). (**F**) Multiple regression analysis demonstrating how the integrated data from 4 antioxidant assays can be used to predict sperm count (*r*^2^ = 0.34; *p* < 0.01). (**G**) Multiple regression analysis demonstrating how the antioxidant assays can be used to predict seminal MDA concentration (*r*^2^ = 0.43; *p* < 0.001). (**H**) Multiple regression analysis demonstrating how the antioxidant assays can be used to predict sperm DNA damage (*r*^2^ = 0.31; *p* < 0.01). (**I**) Multiple regression analysis demonstrating how the antioxidant assays can be used to predict mitochondrial ROS generation by spermatozoa (*r*^2^ = 0.27; *p* < 0.01).

A comparison of how the above 4 assays responded to the antioxidant activity of human semen revealed high levels of correlation between the various assays as well as significant differences. Examination of the total amount of antioxidant activity recorded in the assays indicated that the pre-activation electrochemical assay required higher levels of antioxidant activity to register a response than those recorded with the post-activation assay (*p* < 0.001; Figure 10A), even allowing for the fact that different standards (vitamin C and Trolox) were used in these assays. Since the post-activation assay is entirely focused on the scavenging of a pre-formed radical, this difference must reflect the presence of factors in semen with a powerful capacity to impede free radical formation in the pre-activation ABTS assay. The next highest levels of antioxidant activity were recorded with the hydrogen peroxide scavenging assay (*p* < 0.001), suggesting that seminal plasma also contains factors that scavenge this oxidant (such as catalase, peroxiredoxin 6 or the glutathione peroxidase system) that are not captured by the post-activation free radical scavenging assay. The organic peroxide scavenging assay detected significantly less activity than the hydrogen peroxide scavenging assay (*p* < 0.001; Figure 10A), while the post-activation free radical scavenging assay captured the lowest levels of antioxidant activity of all (*p* < 0.001; Figure 10A).

All of the assays were therefore recording different facets of the same fundamental protective mechanism, and, as such, the activities they measured in semen were generally correlated, even though the absolute levels of antioxidant activity they recorded varied greatly (Figure 10B–E). The highest correlation was observed between the post-activation free radical- and hydrogen peroxide-scavenging activities (*r*^2^ = 0.52; *p* < 0.001) while the lowest was between the organic peroxide and pre-activation electrochemical assay (NS). The assay that is optimal for any given situation presumably varies with the nature of the ROS involved and the vulnerabilities of the target cell type. Thus, if the MDA level in unfractionated semen is used as a marker of oxidative stress in male fertility [33,34], then the hydrogen peroxide scavenging assay was found to be the most highly correlated with this parameter (*r*^2^ = 0.17; *p* < 0.05). Alternatively, if sperm count or progressive motility is selected as a variable reflective of semen quality, then the pre-activation electrochemical assay generated the best correlations with the same group of 27 donors (*r*^2^ = 0.23; *p* < 0.05 and *r*^2^ = 0.16; *p* < 0.05, respectively). With mitochondrial ROS generation, it was the organic peroxide assay that dominated (*r*^2^ = 0.21; *p* < 0.05). In order to capture all the information contained within the 4 antioxidant assays in making predictions of human semen quality, we next undertook a series of multiple regression analyses using semen quality biomarkers as the dependent variables. This form of analysis considers to what extent a dependent variable (in this case, some aspect of semen quality, like motility) can be modeled using data from a group of independent variables (in this case, different measurements of antioxidant activity). Using this approach, predictive algorithms could be written using the antioxidant data that exhibited highly significant correlations with seminal characteristics related to oxidative stress including sperm count (*r*^2^ = 0.34; *p* < 0.01; Figure 10F), seminal MDA (*r*^2^ = 0.43; *p* < 0.001; Figure 10G), sperm DNA damage (*r*^2^ = 0.31; *p* < 0.01; Figure 10H) and mitochondrial ROS generation (*r*^2^ = 0.27; *p <* 0.01; Figure 10I).

## 4. Discussion

The purpose of this study was to develop a series of rapid assays that could be used to measure different aspects of antioxidant activity, using the ABTS•^+^ radical as a common denominator. In the conventional TAC assay, a variety of strategies are used to oxidize ABTS, including metmyoglobin and hydrogen peroxide, potassium persulphate, manganese dioxide and 2,2′-azobis (2-amidopropane) hydrochloride [35,36,37]. The trouble with all these approaches is there will inevitably be some interference with the assessment of antioxidant activity due to the oxidizing system (hydrogen peroxide, persulphate, peroxyl radical, etc.) used in the reaction mixture. A solution to this problem is to use an electrochemical cell to generate the ABTS•^+^ radical in the anodic chamber [38]. This system has the advantage that no extraneous compounds are added to the reaction mixture, and thus the reaction vessel contains only ABTS/ABTS•^+^ and the material under testing. The use of an electrochemical cell also has the advantage that the reducing environment provided by the cathodic chamber does not allow ABTS•^+^ formation, and thus can be used as a convenient control for the impact of sample turbidity on the absorption recorded at 734 nm.

We have exploited this basic principle to develop a modified system that can be used to detect antioxidant activity using either a post-activation or pre-activation approach to focus the assay on free radical scavenging or the disruption of free radical formation, respectively. We have also adapted the ABTS assay to measure hydrogen peroxide- and organic peroxide-scavenging activities employing combinations of either horseradish peroxidase/hydrogen peroxide or hematin/cumene hydroperoxide to induce ABTS activation. We have then compared the antioxidant activities recorded with these assays using a range of putative antioxidant compounds and examined the potential application of these systems in assaying the antioxidant activity of complex biological materials, using human semen as the exemplar.

Many of the antioxidants assessed showed a significant level of antioxidant activity in all assays, confirming their capacity to scavenge a variety of reactive oxygen species and impede free radical formation. Vitamin C, glutathione, N-acetyl cysteine, hypotaurine and melatonin all fell into this category. The polyphenols, EGCG and resveratrol, were also shown to be extremely powerful antioxidants in all versions of the ABTS assay, again indicating their powerful capacity to scavenge ROS and suppress free radical formation. However, several of the putative antioxidants tested appeared to express surprisingly little activity in these assays. Myoinositol, for example, is an important cellular metabolite that is synthesized from glucose and plays an important role in cell biology by serving as a substrate for the synthesis of phosphatidylinositol. This compound is often reputed to be an antioxidant and has, for example, been incorporated into commercial in vitro fertilization media on this basis [39,40]. While there is clear evidence that myoinositol is a valuable medium supplement for both embryos and spermatozoa [39,40,41,42,43], the chemical evidence provided in previous publications [44] as well as the current article indicates that this compound is actually a very weak antioxidant in vitro. Myoinositol must therefore be achieving its antioxidative action via indirect mechanisms, such as the regulation of intracellular calcium [43] or promotion of PI3 kinase/AKT activity [45,46].

Other antioxidants found to exhibit little antioxidant activity in this study such as taurine, spermine and carnitine have previously been found to make little contribution to the antioxidant properties of human semen [31]. Even high doses of carnitine and spermine (100 mM) failed to register significant activity in the post-activation free radical scavenging assay, in keeping with the low activity recorded for these putative antioxidants reported by Rhemrev et al. [31]. Increasing the pH of the assay from 4.8 to 7.4 was investigated to determine if such a strategy might facilitate the deprotonation of hydroxyl groups and facilitate electron transfer, thereby increasing antioxidant activity. However, this strategy proved ineffective with both carnitine and myoinositol (Figure S1, Supplementary Materials). So, while these agents clearly possess antioxidant activity, the major mechanisms by which they achieve their protective effects are not primarily due to ROS scavenging, but rather depend on their abilities to induce indirect antioxidant effects such as the suppression of ROS generation, the stabilization of mitochondrial function or the enhanced cellular generation of antioxidant enzymes [47,48,49,50,51]. Similarly, while co-enzyme Q10 is often reputed be a powerful antioxidant, it does not achieve this end by scavenging ROS. Our results align with those obtained by Cervellati and Greco [52] in indicating that CoQ10 (ubiquinone) possesses little intrinsic ROS scavenging activity; it must be reduced to ubiquinol to achieve such an outcome. Thus, coenzyme Q10′s antioxidant protection is probably achieved via a variety of indirect mechanisms, including intracellular conversion to ubiquinol, the activation of Nrf2 and the subsequent induction of antioxidant enzymes [53] or the suppression of ferroptosis [54].

Lipoic acid has been acknowledged as an antioxidant for some considerable period of time [55]. It is known to scavenge hydroxyl radicals, hypochlorous acid and singlet oxygen; however, it does not appear to scavenge hydrogen peroxide or superoxide anion and probably does not scavenge peroxyl radicals [55,56,57]. Like coenzyme Q10, this compound has to be reduced to dihydrolipoic acid to exhibit significant scavenging activity [58]. In a cellular context, the antioxidant properties of lipoic acid are thought to lie in its capacity to chelate transition metals, suppress mitochondrial ROS generation and rejuvenate other antioxidants such as glutathione following reduction [55,59]. In keeping with previous studies, we found that lipoic acid, per se, exhibited no detectable activity in the scavenging of hydrogen peroxide and only modest scavenging activity with respect to cumene hydroperoxide or the ABTS•^+^ radical. However, lipoic acid exhibited significant activity in the pre-activation ABTS•^+^ assay, suggesting that it can disrupt free radical formation, as suggested by Jia et al. [60]. It shares this property with vitamin C, glutathione NAC, hypotaurine, melatonin and, spectacularly, DMSO. Less than 0.001% DMSO was able to significantly suppress ABTS•^+^ formation in the pre-activation assay, despite having no activity as a scavenger of hydrogen peroxide or the ABTS•^+^ radical and little reactivity towards organic peroxides and peroxyl/alkoxyl radicals. These results support the previous observation that DMSO, at extremely low concentrations, can potently inhibit peroxynitrite-mediated DNA strand breakage and hydroxyl radical formation [61]. Interestingly, all of the agents shown to be particularly effective in the suppression of ABTS•^+^ radical formation in the pre-activation assay (vitamin C, glutathione, hypotaurine, N-acetyl cysteine, melatonin, lipoic acid, resveratrol and EGCG) are known inhibitors of hydroxyl radical formation [62,63,64,65,66]. These results not only suggest the possible involvement of hydroxyl radicals in the electrolytic generation of ABTS•^+^, but also resonate with the known generation of hydroxyl radicals at the anodic surface of electrochemical systems [67,68].

In terms of clinical application, we have examined the behavior of the four assays described in this paper (post-activation determination of free radical scavenging activity; pre-activation assessment of the suppression of free radical formation; hydrogen peroxide scavenging; organic peroxide scavenging) in the analysis of human semen samples. Human semen is acknowledged to be an extremely rich source of antioxidant activity, deficiencies in which have been repeatedly linked to male infertility, encouraging speculation that antioxidant therapy might be useful in the treatment of this condition. Although positive results have been obtained with antioxidant treatments in animal models of male infertility associated with oxidative stress [69], the results of clinical studies have been largely inconclusive [15,70]. One of the major reasons for such confusion is that patients have not been selected for antioxidant treatment on the basis of oxidative stress [16]. Giving antioxidants to patients who are not antioxidant deficient runs the risk of creating a state of reductive stress, which can be just as damaging to human semen quality as its oxidative counterpart [17,18]. The major objective in developing this suite of point-of-care antioxidant assays is to allow such clinical assessments of patient redox status to occur, so that antioxidant therapy can be rationally applied. However, this reasoning raises the question, which antioxidant assay to use?

When the individual assays of antioxidant activity were employed to examine their relationship with semen quality, it was found that no single approach was uniformly more successful than any other. Rather, different methods of assessing antioxidant activity reflected different aspects of semen quality. Thus, variations in the hydrogen peroxide scavenging capacity of human semen optimally reflected the ejaculate’s susceptibility to lipid peroxidation. On the other hand, the ability of semen to suppress free radical formation was best correlated with progressive motility and sperm count, while the organic peroxide scavenging properties of unfractionated human semen most accurately reflected the generation of mitochondrial ROS by defective spermatozoa. In order to accommodate the information contained within all four versions of the antioxidant assay, multiple regression equations were established that enabled reasonably accurate predictions of such key semen quality biomarkers as sperm count, semen MDA, sperm DNA damage and mitochondrial ROS generation. Clearly, more extensive studies are required to establish the detailed nature of these relationships and their significance in the clinical diagnosis of male infertility. To this end, the data presented in this article not only establish a suite of novel methods for measuring different attributes of oxidative stress, but also support the notion that the combined output from these assays will be of value in managing the reproductive health of male patients. Critically, this group of assays, which we have trade marked as ‘the RoXsta^TM^ system’, should also find application in a wide range of additional situations where rapid, multifaceted assessments of antioxidant capacity are required.

## 5. Patents

The methodology described in the article is subject to a patent application (PCT/AU2024/050943).

## Data Availability

The original contributions presented in the study are included in the article/supplementary material, further inquiries can be directed to the corresponding author.

## Supplementary Materials

The following supporting information can be downloaded at: https://www.mdpi.com/article/10.3390/antiox13111379/s1, Figure S1: Impact of pH on antioxidant activity; Figure S2: Antioxidant activity in pre-activation assay; Figure S3: Antioxidant activity in the hydrogen peroxide scavenging assay; Figure S4: Antioxidant activity in the organic peroxide scavenging assay.

## References

[1] Spector A. (2000). Oxidative stress and disease. J. Ocul. Pharmacol. Ther..

[2] Donnelly J.K., Robinson D.S. (1995). Free radicals in foods. Free Radic. Res..

[3] Fortune Business Insights Report ID: FBI 100789. https://www.fortunebusinessinsights.com/industry-reports/food-antioxidants-market-100789.

[4] Kizhakekuttu T.J., Widlansky M.E. (2010). Natural antioxidants and hypertension: Promise and challenges. Cardiovasc. Ther..

[5] Violi F., Pastori D., Pignatelli P., Loffredo L. (2014). Antioxidants for prevention of atrial fibrillation: A potentially useful future therapeutic approach? A review of the literature and meta-analysis. Europace.

[6] Vassalle C., Maltinti M., Sabatino L. (2020). Targeting oxidative stress for disease prevention and therapy: Where do we stand, and where do we go from here. Molecules.

[7] Jones R., Mann T., Sherins R. (1979). Peroxidative breakdown of phospholipids in human spermatozoa, spermicidal properties of fatty acid peroxides, and protective action of seminal plasma. Fertil. Steril..

[8] Aitken R.J., Gibb Z., Baker M.A., Drevet J., Gharagozloo P. (2016). Causes and consequences of oxidative stress in spermatozoa. Reprod. Fertil. Dev..

[9] Smith T.B., Dun M.D., Smith N.D., Curry B.J., Connaughton H.S., Aitken R.J. (2013). The presence of a truncated base excision repair pathway in human spermatozoa that is mediated by OGG1. J. Cell Sci..

[10] Leclerc P., de Lamirande E., Gagnon C. (1997). Regulation of protein-tyrosine phosphorylation and human sperm capacitation by reactive oxygen derivatives. Free Radic. Biol. Med..

[11] Aitken R.J., Harkiss D., Knox W., Paterson M., Irvine D.S. (1998). A novel signal transduction cascade in capacitating human spermatozoa characterised by a redox-regulated, cAMP-mediated induction of tyrosine phosphorylation. J. Cell Sci..

[12] Koppers A.J., De Iuliis G.N., Finnie J.M., McLaughlin E.A., Aitken R.J. (2008). Significance of mitochondrial reactive oxygen species in the generation of oxidative stress in spermatozoa. J. Clin. Endocrinol. Metab..

[13] Tosic J., Walton A. (1950). Metabolism of spermatozoa. The formation and elimination of hydrogen peroxide by spermatozoa and effects on motility and survival. Biochem. J..

[14] Vatannejad A., Tavilani H., Sadeghi M.R., Karimi M., Lakpour N., Amanpour S., Shabani Nashtaei M., Doosti M. (2019). Evaluation of the NOX5 protein expression and oxidative stress in sperm from asthenozoospermic men compared to normozoospermic men. J. Endocrinol. Investig..

[15] de Ligny W., Smits R.M., Mackenzie-Proctor R., Jordan V., Fleischer K., de Bruin J.P., Showell M.G. (2022). Antioxidants for male subfertility. Cochrane Database Syst. Rev..

[16] Aitken R.J. (2021). Antioxidant trials-the need to test for stress. Hum. Reprod. Open.

[17] Sadeghi N., Boissonneault G., Tavalaee M., Nasr-Esfahani M.H. (2023). Oxidative versus reductive stress: A delicate balance for sperm integrity. Syst. Biol. Reprod. Med..

[18] Korge P., Calmettes G., Weiss J.N. (2015). Increased reactive oxygen species production during reductive stress: The roles of mitochondrial glutathione and thioredoxin reductases. Biochim. Biophys. Acta.

[19] Miller N.J., Rice-Evans C.A. (1997). Factors influencing the antioxidant activity determined by the ABTS^+^ radical cation assay. Free Radic. Res..

[20] Biggers J.D., Whitten W.K., Whittingham D.G., Daniels J.C. (1971). The culture of mouse embryos in vitro. Methods in Mammalian Embryology.

[21] Gallegos G., Ramos B., Santiso R., Goyanes V., Gosálvez J., Fernández J.L. (2008). Sperm DNA fragmentation in infertile men with genitourinary infection by Chlamydia trachomatis and Mycoplasma. Fertil. Steril..

[22] Gérard-Monnier D., Erdelmeier I., Régnard K., Moze-Henry N., Yadan J.C., Chaudière J. (1998). Reactions of 1-methyl-2-phenylindole with malondialdehyde and 4-hydroxyalkenals. Analytical applications to a colorimetric assay of lipid peroxidation. Chem. Res. Toxicol..

[23] Salah N., Miller N.J., Paganga G., Tijburg L., Bolwell G.P., Rice-Evans C. (1995). Polyphenolic flavanols as scavengers of aqueous phase radicals and as chain-breaking antioxidants. Arch. Biochem. Biophys..

[24] Miller N.J., Rice-Evans C.A. (1995). Antioxidant activity of resveratrol in red wine. Clin. Chem..

[25] Williamson G., Plumb G.W., Uda Y., Price K.R., Rhodes M.J. (1996). Dietary quercetin glycosides: Antioxidant activity and induction of the anticarcinogenic phase II marker enzyme quinone reductase in Hepalclc7 cells. Carcinogenesis.

[26] Ozgen M., Reese R.N., Tulio A.Z., Scheerens J.C., Miller A.R. (2006). Modified 2,2-azino-bis-3-ethylbenzothiazoline-6-sulfonic acid (ABTS) method to measure antioxidant capacity of Selected small fruits and comparison to ferric reducing antioxidant power (FRAP) and 2,2′-diphenyl-1-picrylhydrazyl (DPPH) methods. J. Agric. Food Chem..

[27] Córdoba A., Alasino N., Asteasuain M., Magario I., Ferreira M.L. (2015). Mechanistic evaluation of hematin action as a horseradish peroxidase biomimetic on the 4-aminoantipyrine/phenol oxidation reaction. Chem. Eng. Sci..

[28] Ilyasov I.R., Beloborodov V.L., Selivanova I.A., Terekhov R.P. (2020). ABTS/PP decolorization assay of antioxidant capacity reaction pathways. Int. J. Mol. Sci..

[29] Re R., Pellegrini N., Proteggente A., Pannala A., Yang M., Rice-Evans C. (1999). Antioxidant activity applying an improved ABTS radical cation decolorization assay. Free Radic. Biol. Med..

[30] Miller N.J., Rice-Evans C., Davies M.J., Gopinathan V., Milner A. (1993). A novel method for measuring antioxidant capacity and its application to monitoring the antioxidant status in premature neonates. Clin. Sci..

[31] Rhemrev J.P., van Overveld F.W., Haenen G.R., Teerlink T., Bast A., Vermeiden J.P. (2000). Quantification of the nonenzymatic fast and slow TRAP in a post-addition assay in human seminal plasma and the antioxidant contributions of various seminal compounds. J. Androl..

[32] Kalyanaraman B., Mottley C., Mason R.P. (1983). A direct electron spin resonance and spin-trapping investigation of peroxyl free radical formation by hematin/hydroperoxide systems. J. Biol. Chem..

[33] Dorostghoal M., Kazeminejad S.R., Shahbazian N., Pourmehdi M., Jabbari A. (2017). Oxidative stress status and sperm DNA fragmentation in fertile and infertile men. Andrologia.

[34] Moretti E., Cerretani D., Noto D., Signorini C., Iacoponi F., Collodel G. (2021). Relationship between semen IL-6, IL-33 and malondialdehyde generation in human seminal plasma and spermatozoa. Reprod. Sci..

[35] Miller N.J., Sampson J., Candeias L.P., Bramley P.M., Rice-Evans C.A. (1996). Antioxidant activities of carotenes and xanthophylls. FEBS Lett..

[36] Bartosz G., Janaszewska A., Ertel D., Bartosz M. (1998). Simple determination of peroxyl radical-trapping capacity. Biochem. Mol. Biol. Int..

[37] Masood N., Fatima K., Luqman S. (2014). A modified method for studying behavioral paradox of antioxidants and their disproportionate competitive kinetic effect to scavenge the peroxyl radical formation. Sci. World J..

[38] Alonso A.M., Domínguez C., Guillén D.A., Barroso C.G. (2002). Determination of antioxidant power of red and white wines by a new electrochemical method and its correlation with polyphenolic content. J. Agric. Food Chem..

[39] Saleh R., Assaf H., Abd El Maged W.M., Elsuity M., Fawzy M. (2018). Increased cryo-survival rate in ejaculated human sperm from infertile men following pre-freeze in vitro myo-inositol supplementation. Clin. Exp. Reprod. Med..

[40] Osman R., Lee S., Almubarak A., Han J.I., Yu I.J., Jeon Y. (2023). Antioxidant Effects of myo-inositol improve the function and fertility of cryopreserved boar semen. Antioxidants.

[41] Colazingari S., Fiorenza M.T., Carlomagno G., Najjar R., Bevilacqua A. (2014). Improvement of mouse embryo quality by myo-inositol supplementation of IVF media. J. Assist. Reprod. Genet..

[42] Mohammadi F., Varanloo N., Heydari Nasrabadi M., Vatannejad A., Amjadi F.S., Javedani Masroor M., Bajelan L., Mehdizadeh M., Aflatoonian R., Zandieh Z. (2019). Supplementation of sperm freezing medium with myoinositol improve human sperm parameters and protects it against DNA fragmentation and apoptosis. Cell Tissue Bank.

[43] De Luca M.N., Colone M., Gambioli R., Stringaro A., Unfer V. (2021). Oxidative stress and male fertility: Role of antioxidants and inositols. Antioxidants.

[44] Płonka J., Szablińska-Piernik J., Buszewski B., Baranowska I., Lahuta L.B. (2021). Analyses of antioxidative properties of selected cyclitols and their mixtures with flavanones and glutathione. Molecules.

[45] Maag D., Maxwell M.J., Hardesty D.A., Boucher K.L., Choudhari N., Hanno A.G., Ma J.F., Snowman A.S., Pietropaoli J.W., Xu R. (2011). Inositol polyphosphate multikinase is a physiologic PI3-kinase that activates Akt/PKB. Proc. Natl. Acad. Sci. USA.

[46] Koppers A.J., Mitchell L.A., Wang P., Lin M., Aitken R.J. (2011). Phosphoinositide 3-kinase signalling pathway involvement in a truncated apoptotic cascade associated with motility loss and oxidative DNA damage in human spermatozoa. Biochem. J..

[47] Marcote M.J., González-Bosch C., Miralles V.J., Hernández-Yago J., Grisolía S. (1989). Polyamines are sufficient to drive the transport of the precursor of ornithine carbamoyltransferase into rat liver mitochondria: Possible effect on mitochondrial membranes. Biochem. Biophys. Res. Commun..

[48] Condorelli R.A., La Vignera S., Bellanca S., Vicari E., Calogero A.E. (2012). Myoinositol: Does it improve sperm mitochondrial function and sperm motility?. Urology.

[49] Li J.L., Wang Q.Y., Luan H.Y., Kang Z.C., Wang C.B. (2012). Effects of L-carnitine against oxidative stress in human hepatocytes: Involvement of peroxisome proliferator-activated receptor alpha. J. Biomed. Sci..

[50] Cao W., Xu X., Jia G., Zhao H., Chen X., Wu C., Tang J., Wang J., Cai J., Liu G. (2018). Roles of spermine in modulating the antioxidant status and Nrf2 signalling molecules expression in the thymus and spleen of suckling piglets-new insight. J. Anim. Physiol. Anim. Nutr..

[51] Fang T., Zheng J., Cao W., Jia G., Zhao H., Chen X., Cai J., Wang J., Liu G. (2018). Effects of spermine on the antioxidant status and gene expression of antioxidant-related signaling molecules in the liver and longissimus dorsi of piglets. Animal.

[52] Cervellati R., Greco E. (2016). In vitro antioxidant activity of ubiquinone and ubiquinol, compared to vitamin E. Helv. Chim. Acta.

[53] Li L., Du J., Lian Y., Zhang Y., Li X., Liu Y., Zou L., Wu T. (2016). Protective effects of coenzyme Q10 against hydrogen peroxide-induced oxidative stress in PC12 Cell: The role of Nrf2 and antioxidant enzymes. Cell. Mol. Neurobiol..

[54] Liu J., Kang R., Tang D. (2022). Signaling pathways and defense mechanisms of ferroptosis. FEBS J..

[55] Packer L., Witt E.H., Tritschler H.J. (1995). Alpha-lipoic acid as a biological antioxidant. Free Radic. Biol. Med..

[56] Wu M.J., O’Doherty P.J., Fernandez H.R., Lyons V., Rogers P.J., Dawes I.W., Higgins V.J. (2011). An antioxidant screening assay based on oxidant-induced growth arrest in Saccharomyces cerevisiae. FEMS Yeast Res..

[57] Scott B.C., Aruoma O.I., Evans P.J., O’Neill C., Van der Vliet A., Cross C.E., Tritschler H., Halliwell B. (1994). Lipoic and dihydrolipoic acids as antioxidants. A critical evaluation. Free Radic. Res..

[58] Biewenga G.P., Haenen G.R., Bast A. (1997). The pharmacology of the antioxidant lipoic acid. Gen. Pharmacol..

[59] Li D.W., Li G.R., Lu Y., Liu Z.Q., Chang M., Yao M., Cheng W., Hu L.S. (2013). α-lipoic acid protects dopaminergic neurons against MPP+-induced apoptosis by attenuating reactive oxygen species formation. Int. J. Mol. Med..

[60] Jia Z., Zhu H., Vitto M.J., Misra B.R., Li Y., Misra H.P. (2009). Alpha-lipoic acid potently inhibits peroxynitrite-mediated DNA strand breakage and hydroxyl radical formation: Implications for the neuroprotective effects of alpha-lipoic acid. Mol. Cell. Biochem..

[61] Jia Z., Zhu H., Li Y., Misra H.P. (2010). Potent inhibition of peroxynitrite-induced DNA strand breakage and hydroxyl radical formation by dimethyl sulfoxide at very low concentrations. Exp. Biol. Med..

[62] Shi X., Flynn D.C., Porter D.W., Leonard S.S., Vallyathan V., Castranova V. (1997). Efficacy of taurine-based compounds as hydroxyl radical scavengers in silica induced peroxidation. Ann. Clin. Lab. Sci..

[63] Aruoma O.I., Halliwell B., Hoey B.M., Butler J. (1989). The antioxidant action of N-acetylcysteine: Its reaction with hydrogen peroxide, hydroxyl radical, superoxide, and hypochlorous acid. Free Radic. Biol. Med..

[64] Poeggeler B., Reiter R.J., Tan D.X., Chen L.D., Manchester L.C. (1993). Melatonin, hydroxyl radical-mediated oxidative damage, and aging: A hypothesis. J. Pineal Res..

[65] Burkitt M.J., Duncan J. (2000). Effects of trans-resveratrol on copper-dependent hydroxyl-radical formation and DNA damage: Evidence for hydroxyl-radical scavenging and a novel, glutathione-sparing mechanism of action. Arch. Biochem. Biophys..

[66] Yoshioka H., Ohashi Y., Akaboshi M., Senba Y., Yoshioka H. (2001). A novel method of measuring hydroxyl radical-scavenging activity of antioxidants using γ-irradiation. Free Radic. Res..

[67] Ghimire B., Lee G.J., Mumtaz S., Choi E.H. (2018). Scavenging effects of ascorbic acid and mannitol on hydroxyl radicals generated inside water by an atmospheric pressure plasma jet. AIP Adv..

[68] Xie J., Zhang C., Waite T.D. (2022). Hydroxyl radicals in anodic oxidation systems: Generation, identification and quantification. Water Res..

[69] Gharagozloo P., Gutiérrez-Adán A., Champroux A., Noblanc A., Kocer A., Calle A., Pérez-Cerezales S., Pericuesta E., Polhemus A., Moazamian A. (2016). A novel antioxidant formulation designed to treat male infertility associated with oxidative stress: Promising preclinical evidence from animal models. Hum. Reprod..

[70] Smits R.M., Mackenzie-Proctor R., Yazdani A., Stankiewicz M.T., Jordan V., Showell M.G. (2019). Antioxidants for male subfertility. Cochrane Database Syst. Rev..

