# Supplementary material for: Towards the Development of Novel, Point-of-Care Assays for Monitoring Different Forms of Antioxidant Activity: The RoXstaTM System
Source: Antioxidants (Basel). 2024 Nov 11;13(11):1379. doi: 10.3390/antiox13111379 (PMC11591381; doi:10.3390/antiox13111379)
Supplement: Supplementary file 1 [file antioxidants-13-01379-s001.zip › antioxidants-3243302-supplementary.pdf]

Impact of pH on antioxidant activity

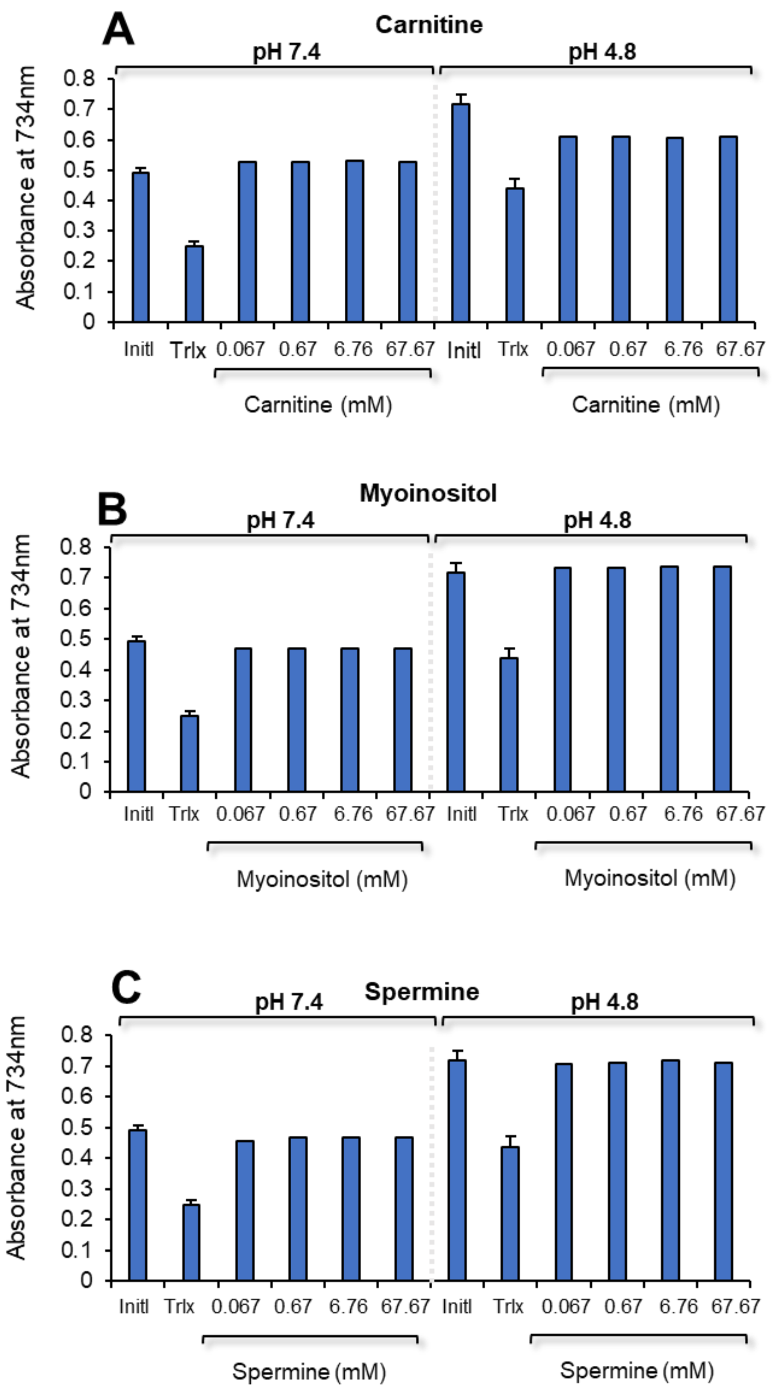

Supplementary Figure S1. Demonstration that elevating the pH at which the post-activation, free radical scavenging assay is conducted, makes no difference to the levels of antioxidant activity observed. Trolox (Trlx) consistently decreased the absorbance associated with the electrochemically generated ABTS$^{\bullet+}$ radical (Initl), however no dose-dependent antioxidant activity was observed at pH 4.8 or 7.4 in response to (**A**) carnitine, (**B**) myoinositol and (**C**) spermine. These compounds therefore appear to possess no significant radical scavenging activity in this antioxidant assay, although the overall rates of absorbance were significantly higher at the lower pH (*$p < 0.001$*).

Antioxidant activity in pre- activation assay

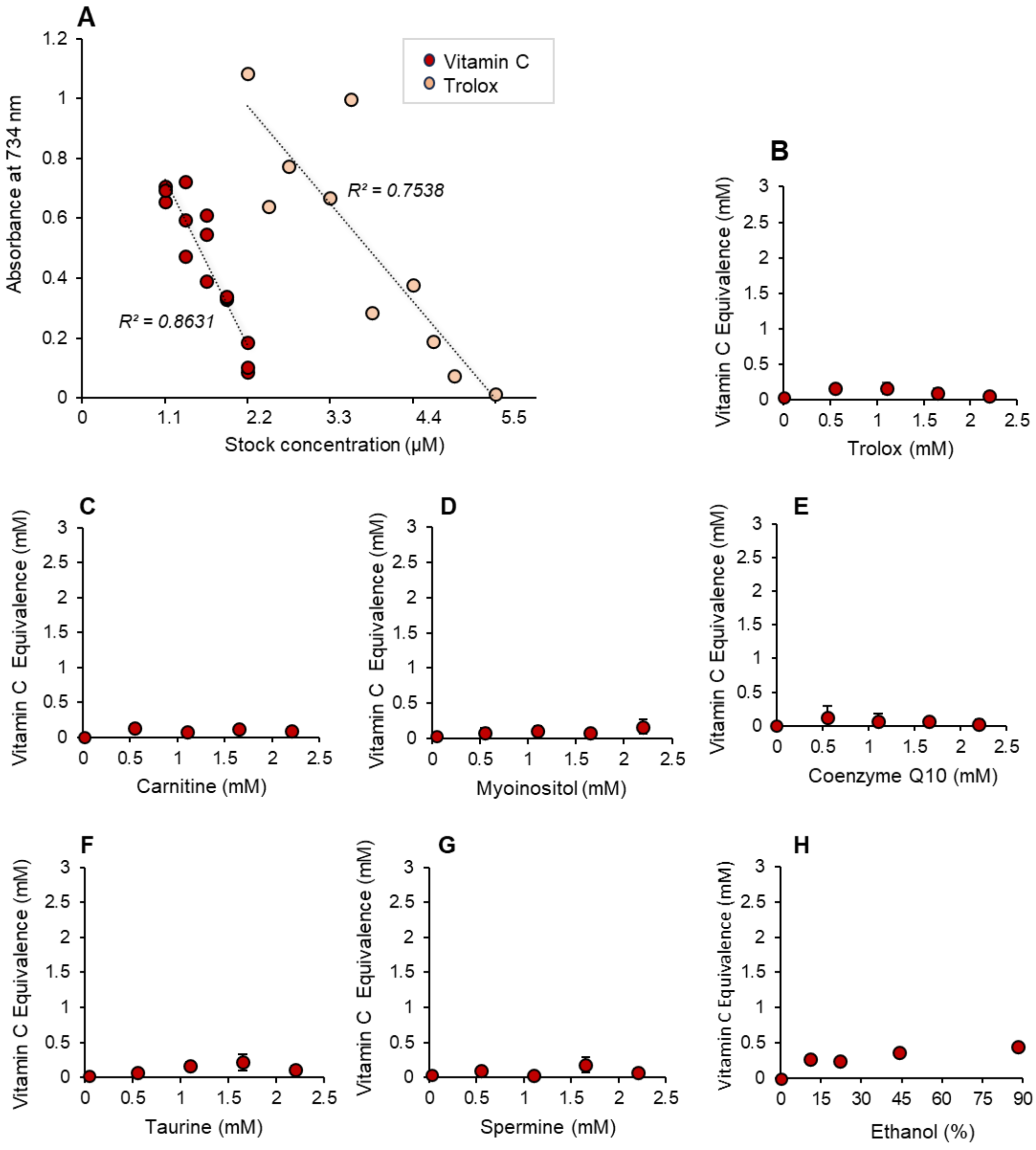

Supplementary Figure S2. Analysis of antioxidant activity with the pre-activation, electrochemical ABTS assay. (**A**) A comparison of vitamin E and Trolox revealed that the pre-activation antioxidant assay is particularly sensitive to vitamin C, whereas Trolox was both less active and gave more variable results. The stock solution was diluted 55-fold in the assay giving a range of final concentrations from 20-100 µM. Given the variability observed with Trolox, vitamin C was selected as the standard for this assay. Comparison of compounds in the antioxidant panel revealed a low level of activity for (**B**) Trolox as well as (**C**) carnitine, (**D**) myoinositol, (**E**) coenzyme Q10, (**F**) taurine, (**G**) spermine, and (**H**) ethanol. **B**-**H.** Data represent mean ± SE of 3 independent assessments.

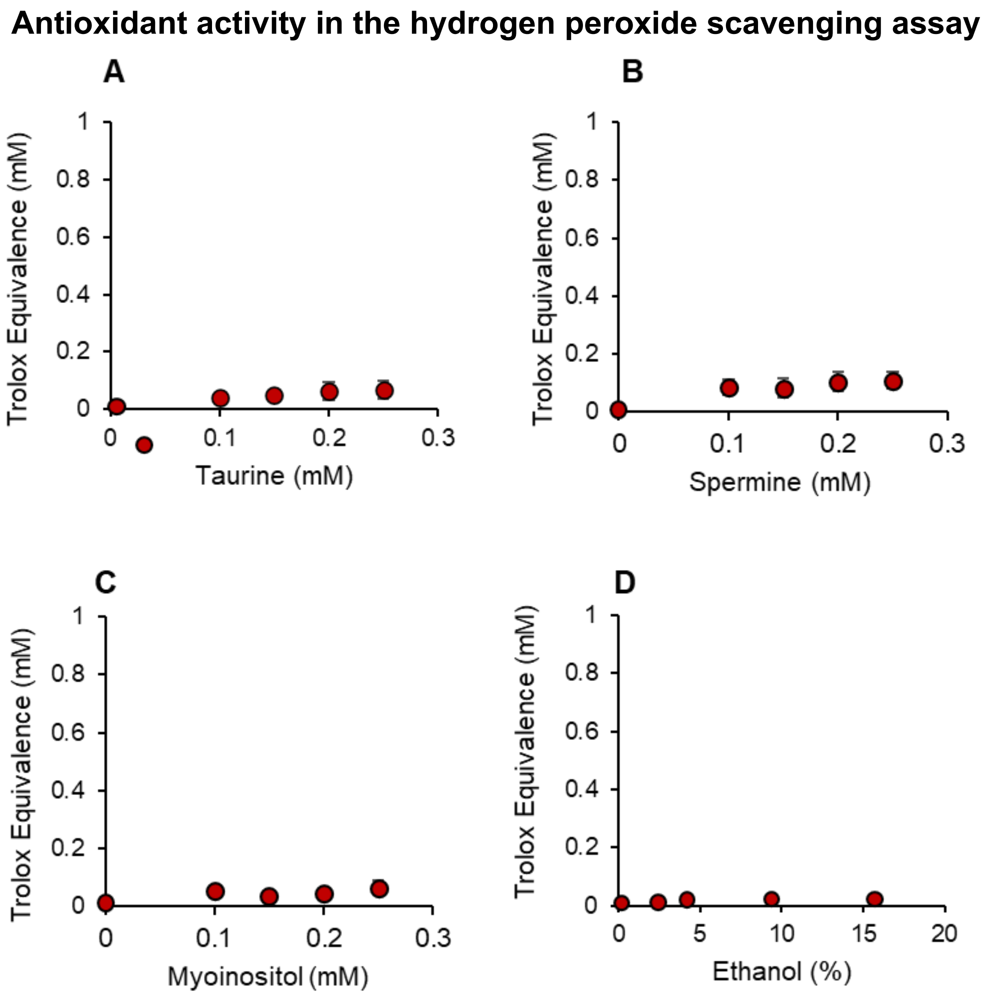

Supplementary Figure S3. Analysis of antioxidant activity with the hydrogen peroxide scavenging system. None of the compounds referenced in this figure exhibited significant activity including (**A**) taurine, (**B**)spermine, (**C**) myoinositol and (**D**) ethanol**.** Data represent mean ± SE of 3 independent assessments.

Antioxidant activity in the organic peroxide scavenging assay

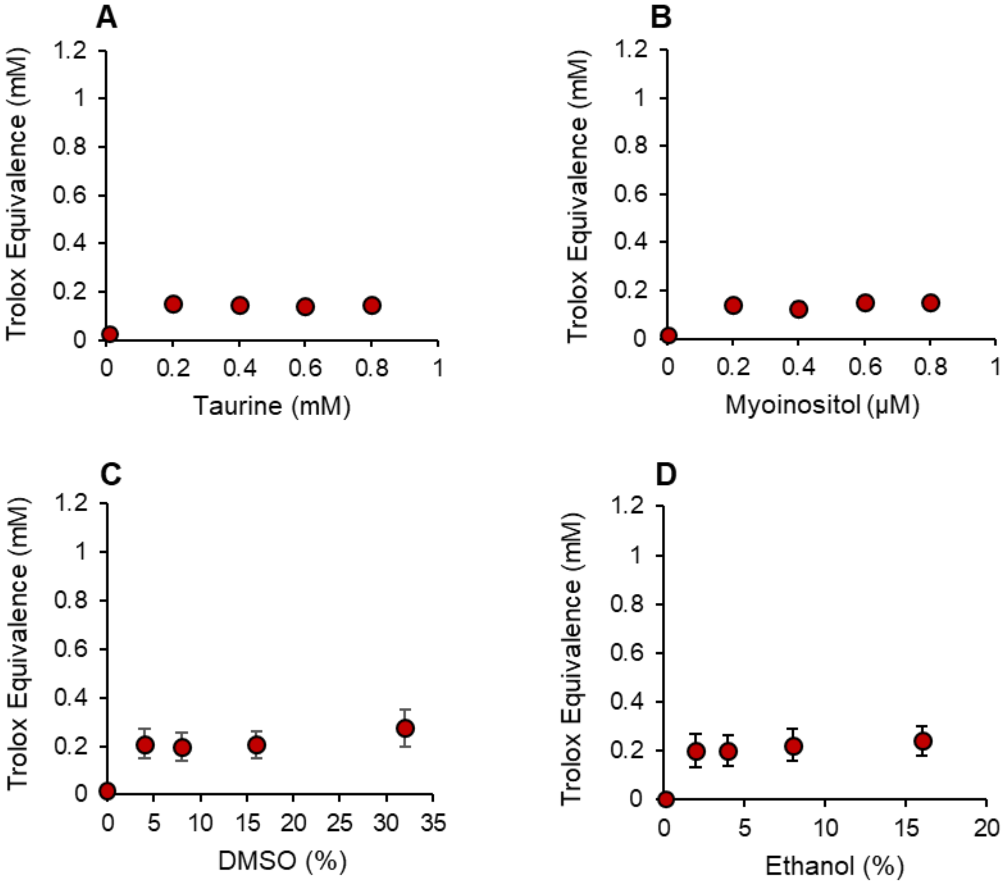

Supplementary Figure S4. Analysis of antioxidant activity with the organic peroxide/peroxide radical scavenging system. All of the compounds referenced in this figure exhibited a low level of activity that was not dose dependent including (**A**) taurine, (**B**) myoinositol, (**C**) DMSO and (**D**) ethanol**.** Data represent mean ± SE of 3 independent assessments.
